# Caudal Neuraxial Blocks for Pain Relief From Pelvic Neuropathy Caused by Extensive Diffuse Large B-cell Lymphoma

**DOI:** 10.7759/cureus.52807

**Published:** 2024-01-23

**Authors:** Amanda Myles, Ammar Toubasi, Elizabeth Soladoye, Narayana Gowda, Joan Morny

**Affiliations:** 1 Internal Medicine, Piedmont Athens Regional, Athens, USA; 2 Anesthesiology, Boston Medical Center, Boston, USA; 3 Anesthesiology and Perioperative Medicine, Augusta University Medical College of Georgia, Augusta, USA; 4 Pulmonary and Critical Care, Piedmont Athens Regional, Athens, USA

**Keywords:** sciatic nerve block, central neuraxial block, neuraxial block, opioid-refractory pain, opioid refractory, refractory cancer pain, cancer pain management, cancer-related neuropathic pain, caudal nerve block

## Abstract

Central neuraxial blocks can be a vital therapeutic tool for neuropathic pain, but they are infrequently implemented for pain management in cancer patients. Upon a literature review, further data on the role or efficacy of central nerve blocks for neuropathic cancer pain would be beneficial. Additionally, evidence-based guidelines and practices are lacking regarding additional interventions for neuropathic pain relief, a common manifestation of cancer burden. Here, we report the case of a 29-year-old male patient who presented in the ED with intractable neuropathic pain from extensive diffuse large B-cell lymphoma. The patient demonstrated left lower extremity pain, fevers, chills, and tenderness with erythema over the site of his port-a-catheter on his chest. The patient was also hypotensive, despite IV fluid resuscitation. Recent imaging showed a hypermetabolic soft tissue mass in the left upper quadrant of the abdomen. There was also extensive cancer spread in the peripheral pelvis, presacral region, and within multiple sacral foramina, with a secondary perineural spread of the tumor. The patient previously positively responded to a caudal nerve block at an outpatient pain clinic. The patient was admitted to the ICU for three days, and following the resolution of sepsis, the patient received caudal and sciatic nerve blocks on admission day 8. Upon further imaging showing metastasis to the brain, the patient was discharged to inpatient hospice on hospitalization day 10 following a palliative conversation with the patient and family.

## Introduction

Cancer-related pain is challenging and complex for both the patient and the healthcare team. Assessing and treating these patients encompasses a multitude of factors to consider, including patient-specific characteristics such as the patient's nature and location of the cancer, as well as the structures the cancer invades, leading to neuropathic symptoms. Other factors to consider include tried-and-tested pain modalities and available therapeutic options. While most cancer patients obtain satisfactory pain relief from medications following the WHO analgesic ladder, approximately 2% to 5% of patients with advanced cancer still have inadequate pain control with systemic analgesics [1].

We report the case of a unique presentation in a 29-year-old male who presented in the ED with disabling neurogenic symptoms from diffuse B-cell lymphoma, which previously had a positive response to a caudal nerve block. Caudal nerve blocks are central neuraxial blocks used for neuraxial anesthesia, and they can be performed via blind technique or with ultrasound or fluoroscopic guidance. Caudal blocks have had a decreased use in obstetric analgesia and simultaneously have increasingly been utilized in the pediatric population, mainly to avoid general anesthesia for procedures as well as to supplement analgesia in the perioperative period [2]. These blocks are also utilized in perioperative and postoperative analgesia for adults with chronic low back pain, with an overall high success rate. However, the utility of caudal nerve blocks for cancer-related pain is not clearly articulated.

Existing knowledge finds that for cancer pain that is refractory or intolerable to opioid analgesia, interventional techniques including neurolysis, neuraxial infusions, and cordotomies may be useful [3]. A 2015 systematic review of nine randomized controlled trials demonstrated that all included techniques of neuraxial analgesia were weakly favored as interventions for adult patients with cancer-related pain, though this review was noted to include low-quality evidence [4]. Furthermore, a PubMed search using the keywords “caudal nerve block” and “cancer pain” resulted in only one study matching the focused question: a case report describing two cases of end-of-life cancer patients with perineal pain, both of whom had pain relief with an ethanol caudal block [5]. Though this case report shows the efficacy of the caudal blocks, the patients were end-of-life, unlike our patient, who had wanted to continue active treatment. Thus, for our patient, who was immunocompromised with a relapse in his cancer, accessing the patient's caudal space with recurrent caudal blocks was concerning for infection.

## Case presentation

We report the case of a 29-year-old male patient who presented to the ED with complaints of a two-day history of fevers, chills, and pain around the site of his implanted chest port, with a chief complaint of intractable left lower extremity neuropathic pain. The patient had a past medical history of relapsed, refractory stage IV extensive diffuse large B-cell lymphoma of the transverse colon and spread in the peripheral pelvic and pre-sacral regions. He had been diagnosed with diffuse large B-cell lymphoma approximately six months prior to presentation. The patient had completed five cycles of rituximab plus cyclophosphamide, doxorubicin hydrochloride, vincristine, and prednisone (R-CHOP) therapy, which was last given about two months prior to presentation.

**Figure 1:**
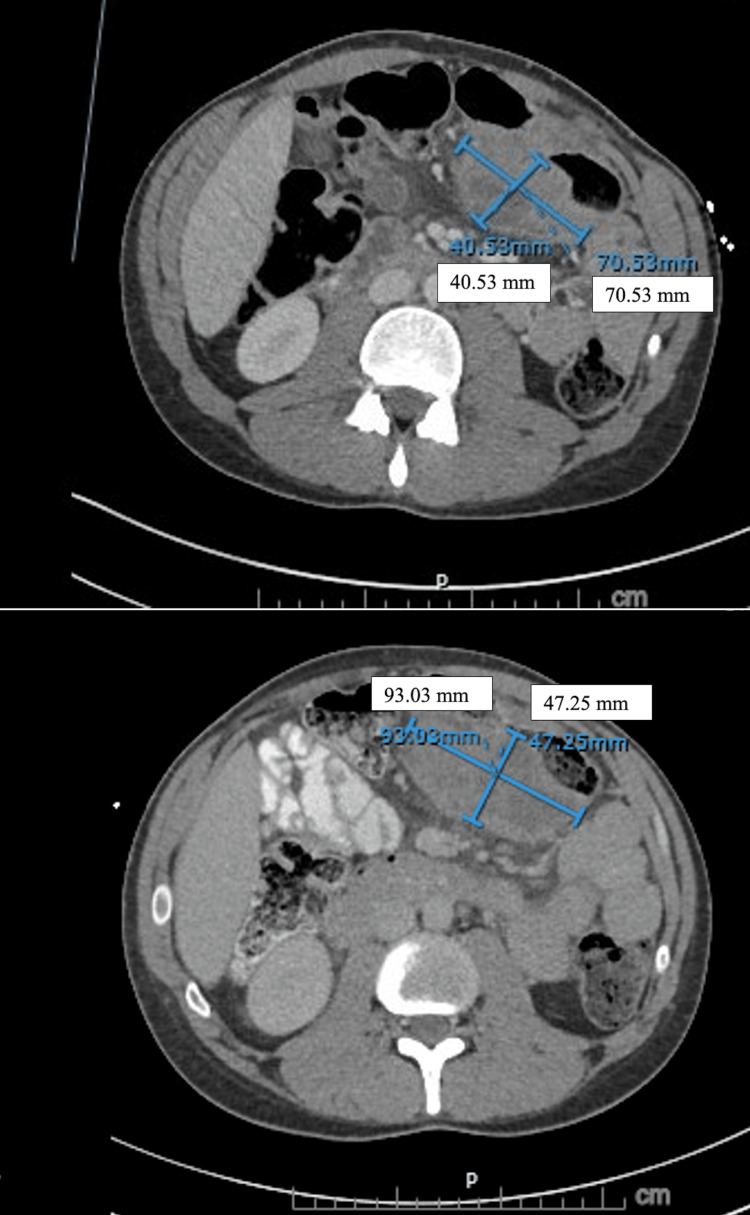
This CT abdomen/pelvis shows rapid disease progression Seen is a mass on the left upper quadrant encasing the segment of the transverse colon. The image on top is of the initial CT, where the mass measures 70.53 mm x 40.53 mm. The image at the bottom is from the follow-up CT performed 20 days after the initial CT, revealing an increase in the mass size (93.03 mm x 47.25 mm).

The patient initially had a positive response on imaging; however, follow-up imaging revealed a recurrence. The PET/CT demonstrated hypermetabolic soft tissue mass in the left upper quadrant of the abdomen, extensive avidity in the peripheral pelvis and presacral region, as well as within multiple sacral foramina with secondary perineural inflammation or perineural spread of tumor (Figure 1). The patient then underwent a CT-guided biopsy of the left upper quadrant abdominal soft tissue mass and lymph node, with pathology and flow suggestive of relapsed lymphoma.

The initial biopsied histopathology image is not available, but the biopsy showed cores of lymphoid tissue with a diffuse infiltrate of atypical cells ranging in size from medium to large, for which the cells of interest were diffusely positive for PAX5 immunostain, negative for CD20 immunostain, and positive for CD3 immunostain in a scattered background of lymphocytes. These findings were consistent with relapsed or persistent large B-cell lymphoma. The patient was subsequently initiated on salvage chemotherapy with rituximab, gemcitabine/oxaliplatin, and then transitioned to polatuzumab/rituximab. Following polatuzumab/rituximab, the patient had plans to proceed with CAR-T cell therapy versus an autologous stem cell transplant. Additionally, the patient has no past surgical history, does not smoke, and has no known drug allergies.

The patient had just been discharged five days before presentation following a six-day hospitalization for intractable left lower extremity pain secondary to left-sided sciatica from the extensive spread of his large B-cell lymphoma. Given the patient's significant perineural spread of the tumor and perineural inflammation, the patient received two sciatic nerve blocks while he was an inpatient. With these peripheral blocks, the patient did not report any noticeable pain relief. Of importance, at a subsequent outpatient pain clinic, the patient received a caudal block with 15 mL of 0.25% bupivacaine and 10 mg dexamethasone, which provided significant pain relief for seven days, which the patient expressed was the longest and most effective relief he had to date.

At the time of this presentation, the patient was alert, oriented, and in acute distress. In addition, the patient provided a numerical rating scale (NRS) score of 10 regarding his left lower extremity. The patient's vitals included a blood pressure of 70/51, a pulse of 140 after receiving two boluses of lactated ringers, a respiratory rate of 20, and a temperature of 99.7 degrees Fahrenheit. As the patient presented with sepsis, this was likely in the setting of a port-a-cath site infection or bacteremia, as the patient was noted to have warm and diaphoretic skin with tenderness and erythema over the site of his port-a-catheter. This catheter was placed six months ago in the left side of his chest for chemotherapy. Due to cancerous perineural extension, the patient also required self-straight catheterizations for urinary retention.

The critical care team was consulted, and the patient was then admitted to the ICU for management of septic shock. Blood cultures returned positive for methicillin-resistant Staphylococcus aureus (MRSA), and the patient was continued on vancomycin. In managing this patient’s cancer-related pain during this hospitalization, the patient continued his home medication regimen of methadone 5 mg every morning, 5 mg at noon, and 10 mg nightly, as well as Dilaudid 4 mg every four hours as needed and tizanidine 4 mg every six hours as needed for muscle spasms. For better pain control, the pain regimen included intravenous Dilaudid 1 mg every four hours as needed. Despite the continued home medication regimen and additional as-needed medications, the patient's pain did not resolve. On the day following admission, the patient had interventional radiology-guided port removal per infectious disease recommendation. The next day, the patient's repeat blood cultures showed no growth, and the patient was transferred out of the ICU to the hospitalist floor and continued antibiotic treatment. Eight days following admission, the patient underwent a fluoroscopy-guided lumbar-sacral caudal neuraxial block with 7.5 mL of 0.25% bupivacaine and 5 mg dexamethasone, as well as a fluoroscopy-guided left sciatic nerve block with 7.5 mL of 0.25% bupivacaine and 5 mg dexamethasone. A subsequent NRS was not provided. The following day, the patient underwent a brain MRI, which showed metastasis. A palliative discussion was held shortly after the brain MRI, and the patient then decided to stop active treatment and proceed with comfort measures.

## Discussion

This case report offers a unique presentation of disabling neurogenic symptoms arising from diffuse B-cell lymphoma invading the perineural structures and emphasizes that central nerve blocks can be a vital therapeutic tool for neuropathic pain. However, they must be properly used in the context of patient characteristics, as there is a risk of complications, including infection. Additionally, this case highlights the benefit of additional literature regarding the efficacy of caudal nerve blocks in cancer pain.

This case provides a clinical vignette showing the short-term benefit of a caudal nerve block, which provided superior pain relief in comparison to prior sciatic nerve blocks. Though the patient only received one caudal neuraxial block before this hospitalization, the administration of dexamethasone along with the anesthetic agent may have contributed to the efficacy of this block, which has been evident in a recent randomized controlled trial [6]. The anti-inflammatory properties of dexamethasone may have provided additional benefits in prolonging the duration of the effect of the nerve block. The administration of steroids needs to be considered in each cancer-related scenario, as these patients are immunosuppressed. Evaluating the current literature in the context of this case presentation highlights gaps in the consensus of knowledge as well as guidelines for physicians. More evidence-based research would be beneficial regarding these blocks for treating neuropathic pain in cancer patients.

This case presentation also brings awareness to in-patient acute pain relief in settings where blocks are contraindicated due to patient characteristics. Of note, our patient, who was admitted in septic shock with septicemia, posed a relative contraindication to any epidural technique given the risk of seeding the epidural space as well as the potential vasodilation resulting in further unfavorable hemodynamics [7,8]. Though our patient presented to the hospital in a state of septic shock, his focus was on the alleviation of his unbearable neuropathic pain. In this instance, how can cancer pain be treated if it is refractory to oral and systemic analgesia? Additionally, acute pain relief with blocks is not an option when there is a lack of skilled staff or equipment to perform blocks. Likewise, numerous complications may arise with nerve blocks, including epidural abscesses as well as local anesthesia toxicity syndrome, which requires experienced physicians to handle such situations.

This report highlights the significance of central neuraxial blocks for intractable pain, a poorly researched and documented clinical therapeutic tool for pain management in cancer patients with neuropathic pain. Rapid recognition and treatment of this complication can improve outcomes and prevent morbidity and mortality in these patients. Special anesthetic considerations should be made when considering the analgesia care plan to understand which patients are appropriate to treat with these blocks. Correspondingly, regardless of the efficacy of central blocks, it is imperative to note that these modalities are not useful for long-term pain management. In addition to neuraxial analgesia, the current evidence of interventional techniques in treating cancer pain includes peripheral nerve blocks, minimally invasive procedures for vertebral pain, sympathetic blocks for abdominal cancer pain, and percutaneous cordotomy. Given these options, the next step for our patient would be a percutaneous cordotomy. However, Kurita et al. found that there is a lack of controlled studies and very few prospective studies on the effects of this treatment for cancer-related pain [9]. While the literature reports that the procedure seems to be safe and effective, the quantity and quality of evidence remain poor. Further research is needed to identify and make available long-term pain management options for chronic pain.

## Conclusions

This case report describes the presentation of a young patient with diffuse B-cell lymphoma refractory to oral pain management with a positive response to a caudal nerve block. Due to septicemia, the patient was managed medically with oral and intravenous opioids, and the patient received caudal and sciatic nerve blocks towards the end of his hospitalization. Later, following the finding of brain metastasis, the patient chose to be managed in an inpatient hospice setting. This report highlights the importance of how central nerve blocks can serve as a vital therapeutic tool, one that is often underutilized in the clinical setting for this purpose. However, the complications of blocks must be considered, especially in immunocompromised patients who are at risk of infection. Further, tailoring the anesthesia care plan can improve outcomes for patients by promoting literature regarding the efficacy of caudal nerve blocks in cancer-related pain and emphasizing the need for other non-invasive therapeutic options.
